# Acupuncture Alleviates Colorectal Hypersensitivity and Correlates with the Regulatory Mechanism of TrpV1 and p-ERK

**DOI:** 10.1155/2012/483123

**Published:** 2012-10-11

**Authors:** Shao-Jun Wang, Hao-Yan Yang, Guo-Shuang Xu

**Affiliations:** ^1^Institute of Acupuncture and Moxibustion, China Academy of Chinese Medical Sciences, Beijing 100700, China; ^2^Basic Medicine, School of Basic Medical Science, Capital Medical University, Beijing 100069, China; ^3^Department of Nephrology, Xijing Hospital, The Fourth Military Medical University, Xi'an 710032, China

## Abstract

Here we used a mouse model of zymosan-induced colorectal hypersensitivity, a similar model of IBS in our previous work, to evaluate the effectiveness of the different number of times of acupuncture and elucidate its potential mechanism of EA treatment. Colorectal distension (CRD) tests show that intracolonic zymosan injection does, while saline injection does not, induce a typical colorectal hypersensitivity. EA treatment at classical acupoints Zusanli (ST36) and Shangjuxu (ST37) in both hind limbs for 15 min slightly attenuated and significantly blunted the hypersensitive responses after first and fifth acupunctures, respectively, to colorectal distention in zymosan treatment mice, but not in saline treatment mice. Western blot results indicated that ion channel and TrpV1 expression in colorectum as well as ERK1/2 MAPK pathway activation in peripheral and central nerve system might be involved in this process. Hence, we conclude that EA is a potential therapeutic tool in the treatment and alleviation of chronic abdominal pain, and the effectiveness of acupuncture analgesia is accumulative with increased number of times of acupuncture when compared to that of a single time of acupuncture.

## 1. Introduction

 IBS is characterized by chronic abdominal pain and altered bowel habits in the relative absence of signs of gastrointestinal inflammation. The pathophysiology of IBS is likely multifactorial [1–4], and the mechanism(s) contributing to discomfort and pain remains unclear, which continues to stimulate the study of peripheral and central nociceptive mechanisms and mediators of hypersensitivity.

 Acupuncture is a procedure in which fine needles are inserted into an individual at discrete points and then manipulated, with the intent of relieving pain. The clinical practice of acupuncture is growing in popularity worldwide. More than 40 disorders have been endorsed by the World Health Organization (WHO) as conditions that can benefit from acupuncture treatment [5–7]. Pain is particularly sensitive to acupuncture. Acupuncture at ST36 and ST37 exerts an antinociceptive effect in rats pain model with PFA injection into the left hind paw [8]. Combined EA at the different acupoints had improving symptoms in IBS patients [9, 10]. Due to previous lack of an ideal experimental animal model suitable for acupuncture research which simulates the human model of chronic visceral hypersensitivity, the mechanism of EA treatment on pain relief in IBS remained unclear. Here we used a mouse model of zymosan-induced colorectal hypersensitivity, a similar model of IBS in Gebhart previous work, and a noninflammatory model of visceral hypersensitivity [11]. In the application of this model in our study, we developed a methodology to assess the effect of different number of times of acupuncture being applied to the same animal which was awake and elucidate its potential mechanism of EA treatment on pain relief in model of visceral hypersensitivity in the mouse.

## 2. Materials and Methods

### 2.1. Animals

 In total, 20 adult male mice (20–30 g, 7 weeks) of C57BL/6 (Taconic, Germantown, NY) were used in this experiment. All procedures were approved by the University of Pittsburgh Institutional Animal Care and Use Committee. 

The mice were divided into four groups as follows.

1. Saline injection via intracolonic route with EA (S + A).
2. Saline injection via intracolonic route without EA (S + C).
3. Zymosan injection via intracolonic route with EA (Z + A).
4. Zymosan injection via intracolonic route without EA (Z + C).

**Figure 1:**
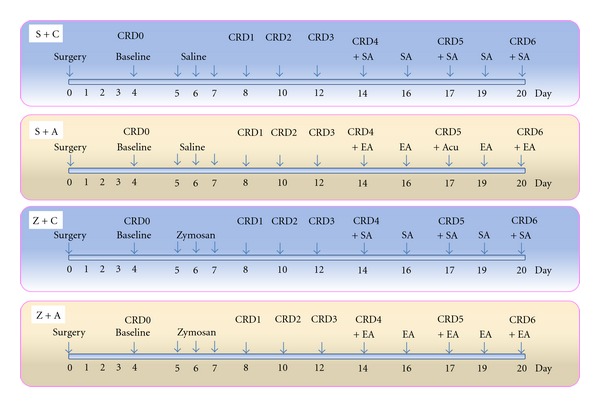
Experimental protocol, 20 mice are used. 5 mice in each group. There are 4 groups. S + C: saline + sham-acupuncture (SA), S + A: saline + EA, Z + C: zymosan + SA, Z + A: zymosan + EA.

Five mice were used in each group. Zymosan induced colorectal hypersensitivity animal model and EA interference was shown by schematic diagram in Figure 1.

### 2.2. Intracolonic Treatments

 Mice were anesthetized with 2% isoflurane (Hospira Inc., Lake Forest, IL). Either 0.2 mL of vehicle (saline) or a suspension of 30 mg/mL zymosan (a protein-carbohydrate cell wall derivative of the yeast *Saccharomyces cerevisiae* in saline; Sigma Chemical Co, St. Louis, MO) was administered transanally via a 22-gauge, 24 mm long stainless-steel feeding needle. Intracolonic treatment with saline or zymosan was performed daily for 3 consecutive days after obtaining baseline response measures to colorectal distension (CRD) before assessing colorectal hypersensitivity.

### 2.3. Electromyographic Electrode Implantation and CRD Testing

 Electromyographic Electrode Implantation was performed at day 0. Following anesthesia, the left abdominal musculature was exposed by incision of the skin, and the bare ends of 2 lengths of Teflon-coated (Cooner Wire Sales, Chatsworth, CA) stainless-steel wire (Cooner Wire Sales, Chatworth, CA) were inserted into the abdominal muscles and secured in place with 5–0 polyglactin sutures (Ethicon, Somerville, NJ). The other wire ends were tunneled subcutaneously to a small incision made on the nape of the neck and externalized for access during testing. Four days was allowed for recovery from surgery before initiating the CRD protocol. On the day of testing, mice were briefly sedated with isoflurane for balloon insertion. Distension balloons were made from polyethylene (length, 1.5 cm; diameter, 0.9 cm) [11] coated with lubricant, inserted transanally until the proximal end of the balloon was 0.5 cm from the anal verge (total balloon insertion 2 cm), and secured to the mouse tail with tape. Mice were placed in a restraint device (manufactured as described previously [12]) inside a sound-attenuating, dark chamber and allowed to recover from isoflurane sedation (30 minutes) before CRD testing. CRD was performed as previously described [12]. Briefly, electromyographic (EMG) activity was recorded for 10 seconds before and during phasic balloon inflation (15, 30, 45, or 60 mmHg) of the colorectum. Each distension lasted 10 seconds, and each pressure was tested 3 times with 5 minutes apart and between distensions. EMG electrode activity was amplified, filtered, rectified, and quantified using Spike 2 software (Cambridge Electronic Design, Cambridge, UK) and recorded on a computer. Responses to CRD were quantified as the total area of EMG activity during balloon inflation minus baseline activity in the 10 seconds prior to distension.

 The first time CRD test was performed on day 4 as a baseline (CRD_0_) and after which saline or zymosan was injected via intracolonic route. Subsequently, the CRD tests were recorded on day 8 (CRD_1_), day 10 (CRD_2_), day 12 (CRD_3_), day 14 (CRD_4_), day 17 (CRD_5_), and day 20 (CRD_6_). EA or SA was performed on day 14, day16, day 17, day 19, and day 20 (Figure 1). Mice were sacrificed immediately after the last CRD test (CRD6), and the fresh tissues of brain (thalamic area), spinal cord (L_5_-S_2_), and colorectum (the lengths 1 cm above anorectal line) were collected.

### 2.4. EA Treatment

 EA was applied by two pair of stainless steel needles (0.25 mm in diameter) inserted bilaterally at a depth of 3 mm into the skin and underlying muscles at ST36 (2 mm lateral to the anterior tubercle of the tibia and 3 mm below the knee joint plus) [13, 14] and ST37 (2 mm lateral to the anterior tubercle of the tibia and 6 mm below the knee joint, minus) (Figure 3. Acupoints). The needles, which were inserted into acupoints, were stimulated by an EA apparatus (#HANS-100A, Nan Jing Ji Sheng Medical Treatment Science and Technology Co., China) with a constant rectangular current of alternating trains of dense-sparse frequency (2/100 Hz, pulse width, 0.2–0.6 msec). This combination of dense-sparse frequency would maximally induce opioid release of met-enkephalin and dynorphin A [15]. Electrical stimulus intensity was set at the threshold for a detectable muscle twitch (approximately 1 mA). The stimulation was delivered for 15 min. For sham EA group, the needle set was same EA group, but no electrical stimulation was applied. CRD tests were performed 30 min after termination of EA.

### 2.5. Western Blot Analysis

Western blot analysis was performed as follows [16]: mice tissues of colorectum, spinal cord, or brain were homogenized on ice in RIPA buffer (50 mol/L Tris-Cl, pH 7.6, 5 mol/L ethylenediaminetetraacetic acid, 150 mol/L NaCl, 0.5% Nonidet P-40, 0.5% Triton X-100) containing protease inhibitor cocktail and phosphatase inhibitor cocktails I/II (Sigma-Aldrich). The homogenate was centrifuged at 12,000 ×g for 30 minutes at 4°C. The supernatant was collected and the protein concentration was measured using the Bradford assay. Twenty micrograms of the sample was separated with 10% polyacrylamide gel blotted on a PVDF film (Millipore Corp). The blotted film was blocked for 2 hours at 4°C in blocking solution (1 × TBS with 5% nonfat milk and 0.02% Tween 20). The blocked film was shaken overnight at 4°C using primary antibodies in blocking solution. Following three times of washing with TBST (1 × TBS with 0.02% Tween 20), the film was shaken for 1 hour at room temperature with peroxidase-conjugated secondary antibody and then washed three times with TBST. Detection was performed using an ECL kit (Santa Cruz Biotech) according to the manufacturer's instructions. The western blots shown are representative of at least three independent experiments.

The antibodies used included the following: anti-TrpV1 (Alomone Labs, #Acc-030) (1 : 800), antiphospho- and antitotal ERK_1/2_ (cell signaling, #9109 and #4695,) (1 : 2000), anti-PGP9.5 (Neuromics, #RA12103) (1 : 20000), anti-*β*-Actin (Sigma, A5316) (1 : 100000), HRP-conjugated IgG secondary antibodies (1 : 2000) (GE Healthcare Life Sciences). All western blot data were analyzed by Image J software.

### 2.6. Statistical Analysis

All CRD test data were expressed as mean ± SEM. Statistical significance of visceromotor response (VMR) values of the area under the curve (AUC) from EMG recording was determined using two way analysis of variance (ANOVA) with repeated-measures via GraphPad Prism (Avenida de la Playa La Jolla California).

All results with *P* values less than 0.05 were considered statistically significant. 

## 3. Results

### 3.1. Zymosan-Induced Visceral Hypersensitivity

 Visceromotor responses (VMR) to colorectal distension were recorded as EMG measurements before (day 4) and after intracolonic administration of saline or zymosan (days 8, 10, and 12), and only on 3 days after acupuncture treatment (days 14, 17, and 20), though EA treatment was administered on days 14, 16, 17, 19, and 20. The measurement of EMG on days 17 had additional assessment value for consecutive EA treatment of 2 days. In addition EMG measurement at days 20 will shed light on the cumulative therapeutic effect of the entire duration of EA therapy. 

 On day 4 (baseline, before any intracolonic treatment or CRD_0_), groups S + C, S + A, Z + C, and Z + A of mice all responded in a graded manner to increasing pressures of colorectal distension. However, visceromotor responses (VMRs) to distension among 4 groups had no significant difference, revealing that all mice from different groups had similar response to pressures of colorectal distension before any intracolonic treatment. To compare VMR to distension overtime, responses within four groups of mice are normalized to 60 mm Hg in subsequent data presentations.

After 3 times of intracolonic injection of saline or zymosan on days 5, 6 and 7, CRD tests were performed on days 8 (CRD_1_), 10 (CRD_2_), and 12 (CRD_3_). 

**Figure 2:**
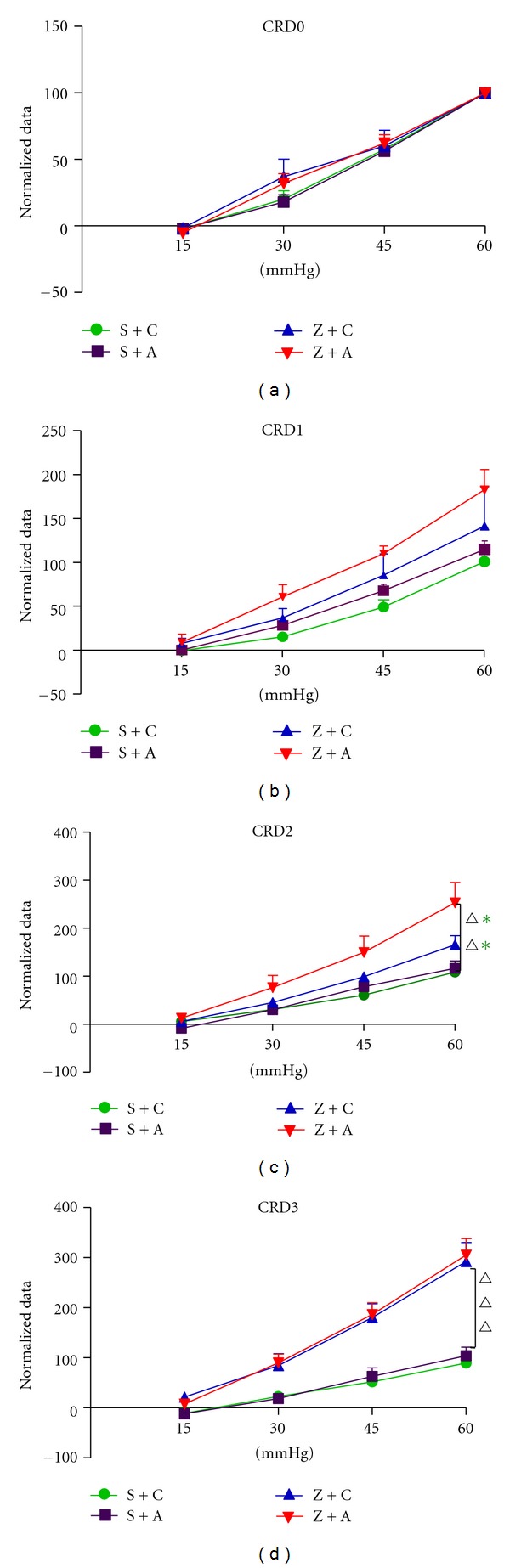
CRD test results before EA. CRD0: responses to colorectal distension in 4 groups before (baseline) saline or zymosan treatment (F3,16 = 0.542, *P* > 0.05); CRD1: responses to colorectal distension in 4 groups after intracolonic treatment with saline or zymosan (F3,16 = 2.593, *P* > 0.05) on day 8; CRD2: responses to colorectal distension in 4 groups after intracolonic treatment with saline or zymosan (F3,16 = 6.021, *P* = 0.006, in Z + C versus S + A and Z + C versus S + C) on day 10; CRD3: Responses to colon distension in 4 groups after intracolonic treatment with saline or zymosan (F3,16 = 15.24, *P* < 0.001, in Z + C versus S + A, Z + C versus S + C, Z + A versus S + A, and Z + A versus S + C) on day 12. Colorectal hypersensitivity did not develop in saline-treated mice. Data are expressed as percentage (mean ± SEM) of the VMR to colorectal distension on the day of baseline testing (day 4) for each animal and normalized to the response to 60 mm Hg distension (100%) to generate stimulus-response functions.

Figure 2 CRD1 showed that intracolonic saline or zymosan treatment did not produce persistent colorectal hypersensitivity in 4 groups of mice. CRD_2_ showed that intracolonic saline treatment did not produce, whereas intracolonic zymosan treatment did produce, persistent colorectal hypersensitivity in group Z + A mice. However, one of the zymosan treatment groups, Z + C, showed increasing of colorectal hypersensitivity but had no significant difference compared with saline treatment group. On day 12, CRD_3_ showed that both saline treatment groups did not, while both zymosan treatment groups did, exhibit persistent colorectal hypersensitivity, suggesting that at least 4–8 days' time period was required for maturing of zymosan-induced colorectal hypersensitivity.

### 3.2. EA Treatment Suppressed Zymosan-Induced Visceral Hypersensitivity in Mice

**Figure 3:**
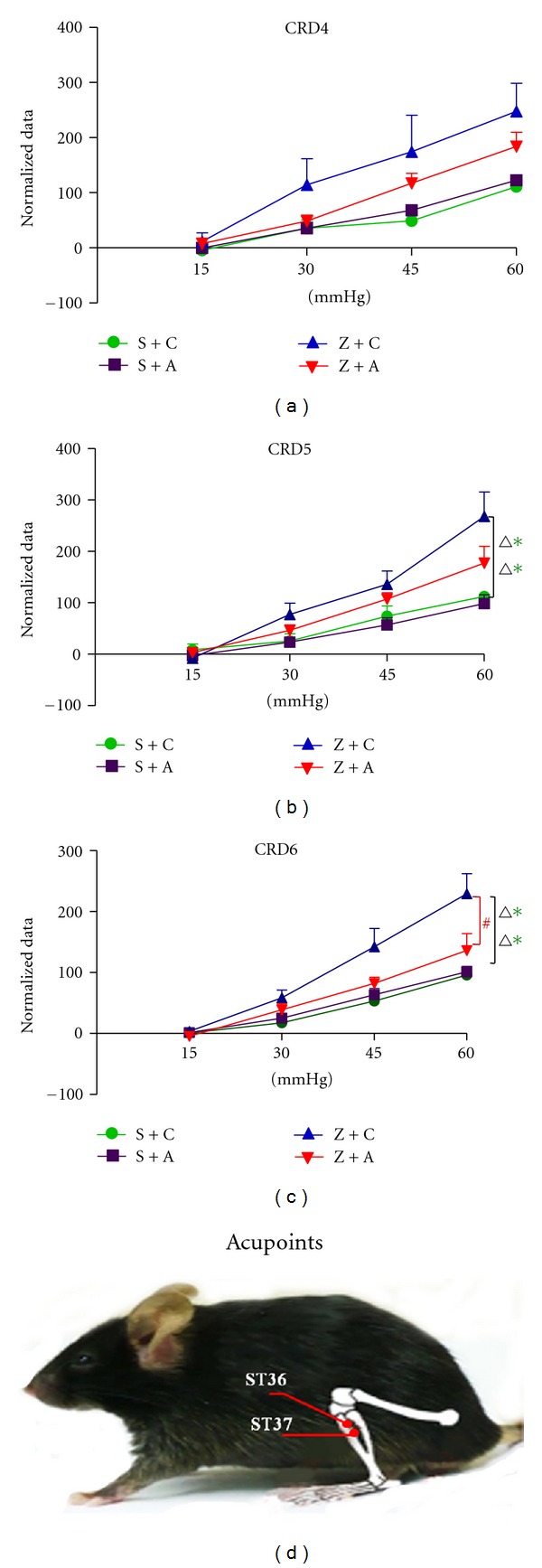
CRD test results after EA. CRD4: responses to colorectal distension in 4 groups after first time EA (F3, 16 = 3.216, *P* = 0.051); CRD5: responses to colorectal distension in 4 groups after third-time EA (F3, 16 = 5.853, *P* = 0.007, in Z + C versus S + A, Z + C versus S + C); CRD6: responses to colorectal distension in 4 groups after fifth-time EA (F3, 16 = 10.541, *P* < 0.001, in Z + C versus S + C, Z + C versus S + A, and Z + C versus Z + A). Acupoints: schematic representation of the ST36 and ST37.

**Figure 4:**
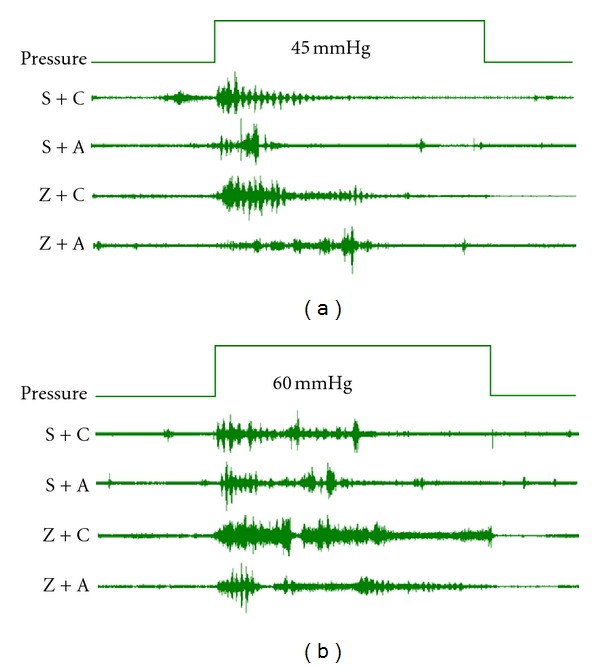
Examples of EMG activity in mice with or without EA. (a) indicates EMG activity results under 45 mmHg; (b) indicates EMG activity results under 60 mmHg.

Figure 3 showed the CRD tests results after acupuncture. CRD4 showed EMG activity in group Z + C was significantly increased than that in group S + C and S + A after first time EA. However, there was no significant difference among the four groups. CRD5 showed EMG activity in group Z + C was significantly increased than those in saline groups S + C and S + A. CRD6 showed EMG activity in group Z + C was significantly increased than that in saline groups S + C and S + A, while the elevation of EMG activity was significantly blunted in group Z + A after fifth time EA, as shown by the EMG results in Figures 4(a) and 4(b). These data demonstrated that EA treatment suppresses zymosan-induced visceral hypersensitivity, which is in agreement with previous studies that EA treatment attenuated chronic visceral hyperalgesia induced by neonatal colonic injection of acetic acid [17]. As expected, the relief of visceral hypersensitivity-induced pain increased with more EA treatments.

### 3.3. EA Blunts Zymosan-Induced Expression of TRPV1

**Figure 5:**
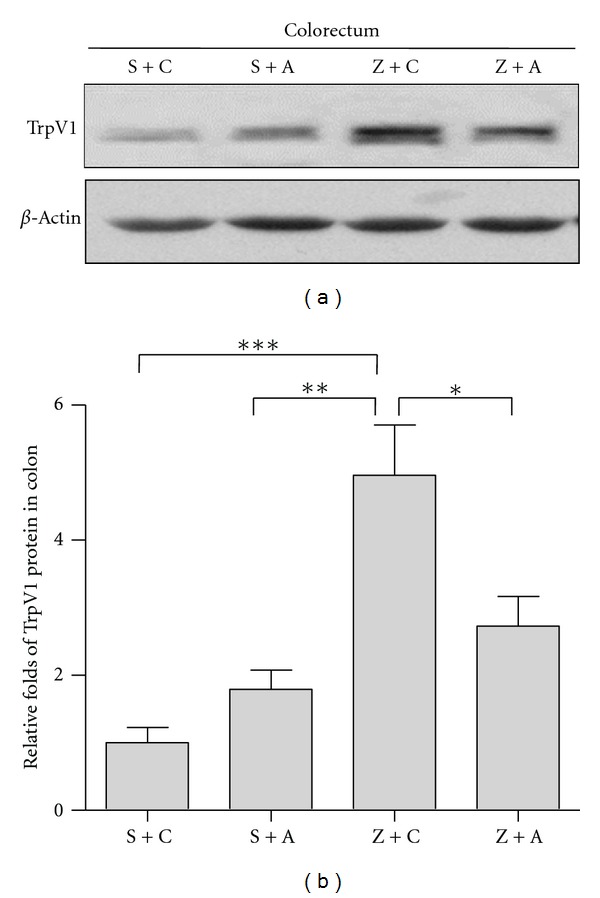
EA blunts zymosan-induced expression of TrpV1. Western blot shows detectable signal of TrpV1 in colon. TrpV1 expression slightly increased in response of EA in group S + A (S + A versus Z + C, *P* < 0.01), while it is dramatically induced by zymosan injection(S + C versus Z + C, *P* < 0.001). EA obviously compromises zymosan-induced upregulation of TrpV1(Z + A versus Z + C, *P* < 0.05) (#Acc-030, Alomone Labs, 150KD, delusion: 1 : 800).

 Western blot analysis showed that there was a detectable signal of transient receptor potential vanilloid 1(TrpV1) in the colorectum (Figure 5). TrpV1 expression was only slightly increased in response to acupuncture in group S + A, while it was dramatically induced by zymosan injection. EA obviously compromises zymosan-induced upregulation of TrpV1. The Z + C group displayed an obviously higher expression of TrpV1 when compared with the S + C group, by 80%. However, the TrpV1 level in S + A group manifested 44.43% up-regulation, when compared with the S + C group. This in turn demonstrated that EA greatly compromised zymosan-induced up-regulation of TrpV1 by 87.58%. 

These data suggested that TrpV1 is involved in zymosan-induced visceral hypersensitivity and EA suppressed visceral hypersensitivity. We found that TrpV1 protein is detectable only in colorectal tissue by Western blot. EA treatment alone can slightly induce TrpV1 in S + A group.

### 3.4. EA Blunts Zymosan-Induced ERK1/2 Activation

**Figure 6:**
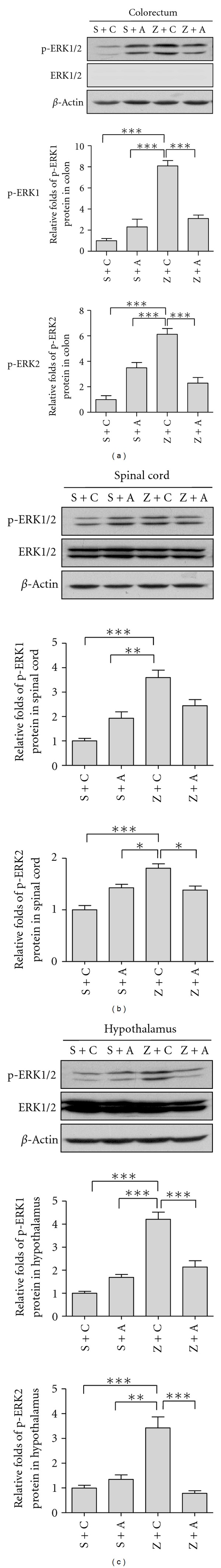
EA blunts zymosan-induced ERK1and ERK2 activation. ERK1 and ERK2 activation is presented by phosph-ERK1 and ERK2 versus their related total ERK1 and ERK2. ERK1 and ERK2 activation is slightly induced by EA in group S + A, while it is dramatically induced by zymosan injection. Zymosan-induced ERK1 and ERK2 activation is obviously compromised by EA in colorectum (*P* < 0.001), spinal cord (*P* < 0.05), and hypothalamus (*P* < 0.001). EA in alleviating hypersensitivity is probably associated with external signaling (p-ERK1/2: #9109, cell signaling, 42/44 KD, delusion: 1 : 2000; ERK1/2: #4695, cell signaling, 42/44 KD, delusion: 1 : 2000).

 Extracellular signal-regulated kinase1/2 (ERK1/2) is protein kinase signaling pathways. ERK1/2 activation is presented by phosph-ERK1/2 (p-ERK) versus their related total ERK1/2 (Figure 6). ERK1, and ERK2 activation was induced by acupuncture in group S + A by 56.59% and 71.32%, 48.27% and 29.98%, 40.79%, and 25.89% when compared with group S + C in colorectum, spinal cord, and hypothalamus, respectively. It was dramatically increased in Z + C group by 87.63% and 83.68%, 72.22% and 44.68%, 76.28% and 70.83% compared with the S + C group in colorectum, spinal cord, and hypothalamus, respectively. However, zymosan-induced ERK1and ERK2 activation is obviously compromised by EA. The Z + A group displayed a reduction of p-ERK expression by 160.32% and 167.19%, 47.34% and 30.63%, 96.98% and 337.05% when compared with the Z + C group in colorectum, spinal cord and hypothalamus, respectively. These data suggested that the processing of EA analgesia information occurs at central as well as at peripheral sites in the mice with zymosan induction.

### 3.5. Zymosan Could Not Induce PGP9.5 Activation

 Protein gene product (PGP) is neuron specific protein, structurally and immunologically distinct from neuron specific enolase. PGP9.5 is in neurones and nerve fibers at all levels of the central and peripheral nervous system. As a pan-neuronal marker, it may also be expressed by nonneural cells such as enteroendocrine cells.

**Figure 7:**
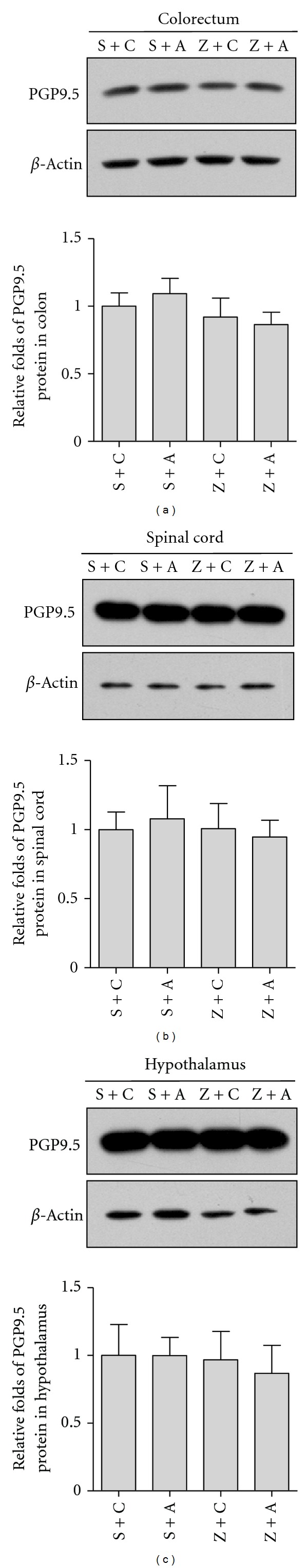
Zymosan could not induce PGP9.5 activation. There are no big differences in PGP9.5 expression among the 4 groups in colorectum, spinal cord, and hypothalamus, respectively. (#RA12103, Neuromics, 27KD, delusion: 1 : 20000).

Therefore we determined that PGP9.5 is involved in colorectal distention process. The results showed that PGP9.5 (Figure 7) is predominantly expressed in colorectum, spinal cord, and hypothalamus. However, there is no significant difference in PGP9.5 expression among the 4 groups in colorectum, spinal cord, and hypothalamus, respectively.

## 4. Discussion

 The principal findings were as follows: (1) zymosan-induced elevation of visceromotor responses to colorectal distension is significantly attenuated by EA, suggesting that EA blunts zymosan-produced colorectal hypersensitivity; (2) visceral hypersensitivity-related molecules' results showed that TrpV1 expression in colorectum is slightly increased in response of EA in mice without zymosan injection. Zymosan dramatically induces TrpV1 expression, and the induction of TrpV1 is greatly blunted by EA in colorectum. Consistently, ERK1/2 phosphorylation in colorectum, spinal cord, and hypothalamus is also slightly increased in response to EA in mice without zymosan injection and is dramatically induced by zymosan injection. EA greatly compromised zymosan-induced ERK1/2 phosphorylation. However, after Western blot analysis, we did not detect any difference in PGP9.5 expression among the 4 groups in colorectum, spinal cord, and hypothalamus. These findings support the fact that the processing of EA analgesia information occurs at central as well as at peripheral sites in the mice with zymosan induction.

### 4.1. This Animal Model Suits for Acupuncture Research

 Acupuncture and EA are widely used in pain relief in clinic [18, 19] and in relieving inflammation-induced hypersensitivity in rat models [8, 20]. However, in the treatment of visceral hypersensitivity, the use of acupuncture once is often not enough. The use of an animal model that can be assessed for cumulating effect of different number of times of acupuncture in the same animal, which is awake, is important in the research evaluation of acupuncture treatment in IBS and the elucidation of the involved mechanism(s). In the current study, we developed a noninflammatory colorectal hypersensitivity model in which an intracolonic treatment with zymosan, a protein-carbohydrate cell wall derivative of the yeast *Saccharomyces cerevisiae*, was capable of producing a robust and chronic behavioral hypersensitivity to colorectal distention [21, 22]. It is noteworthy that in this animal model there were two electrodes in between the two ears of each mouse; the other ends of these two electrodes were fixed on the abdominal muscles; hence, we can use this animal model for real-time monitoring of effect of acupuncture on visceral hypersensitivity in the awaked animal by EMG, with continuous observation of the effect of different number of times of acupuncture in vivo in the same animal. This has not been achieved in other animal models.

### 4.2. EA Treatment Suppressed Zymosan-Induced Visceral Hypersensitivity in Mice

The EA procedures may stimulate the somatic afferent nerves innervating the skin and muscles of the body, which are thought to be specific points that reflect visceral conditions and organs [23]. We found that EA treatment significantly reduced and suppressed EMG responses to colorectal distention in zymosan-induced noninflammatory colorectal hypersensitivity (Figures 4(a) and 4(b)) in the present study. Moreover, the effectiveness of acupuncture analgesia increases with the increase in number of times of acupuncture (Figure 3 CRD4, CRD5, and CRD6), indicating that EA had an analgesic effect in this model. These findings clearly showed the effectiveness of EA at the ST36 and ST37 acupoints on anti-non-inflammatory pain.

### 4.3. EA Blunts Zymosan-Induced Expression of TrpV1

The transient receptor potential vanilloid 1 (TrpV1) is an important protein for ligand-gated ion channels formation in peripheral sensory neurons [24, 25] and is believed to play a pivotal role in visceral hypersensitivity [26]. TrpV1 upregulation was found in colorectal samples from patients with inflammatory bowel disease and Crohn's disease [27], as well as in sensory fibers from patients with rectal hypersensitivity [28]. This present study concurs with our previous work [21] that TrpV1 was involved in zymosan-induced an noninflammation-independent colorectal hypersensitivity. Interestingly, EA treatment compromised zymosan-induced TrpV1 up-regulation in colorectum, suggesting that EA, partially through regulating TrpV1 expression, reduces zymosan-induced colorectal hypersensitivity. However, when compared with complete blockage of EMG activity in mice with zymosan injection, EA treatment only partially retarded TrpV1 protein level, suggesting that other molecules or central nerves system is likely to be involved in the processing of acupuncture analgesia. 

### 4.4. EA Blunts Zymosan-Induced ERK1/2 Activation

ERK, activated by neurotrophins or neuronal activity in the central or peripheral nervous system, plays an essential role in the generation and maintenance of inflammation-induced hyperalgesia by regulating nociceptive activities in primary sensory pathways [29, 30]. Increased phosphorylation of ERK1/2 has been observed in rat spinal cord dorsal horn neurons in response to noxious stimulation of the peripheral tissue or electrical stimulation to the peripheral nerve [31, 32]. Moreover, intrathecal injection of MEK/ERK inhibitor U_0126_ or PD_98059_ alleviated pain behaviour induced by inflammation of the hind paw [32] or viscera [31], suggesting a prominent role of ERK in the regulation of peripheral inflammation. We found that both EA treatment and zymosan injection alone greatly induced ERK1/2 phosphorylation in colorectum, spinal cord, and brain, suggesting that ERK1/2 MAP kinase pathway is involved in transduction of pain signal in peripheral as well as in central nervous system. However, ERK1/2 phosphorylation was obviously suppressed both in colorectum and in spinal cord by EA treatment. Importantly, it was completely blocked by EA treatment in hypothalamus. These results highly suggested that EA treatment might have an increased blocking capacity for pain signal from peripheral to central nervous system in colorectal hypersensitivity mouse model via blocking of ERK1/2 MAPK activation, while it has no effects on pain signal transduction in CNS or even enhancing signal of peripheral nervous system in normal control mice. 

### 4.5. Zymosan Could Not Induce PGP9.5 Activation

 PGP9.5 nerve fibres are believed to be involved in IBS [33]. It was detected in the myenteric plexus, but PGP 9.5-immunoreactive cell bodies did not colocalize with TrpV1 [26], suggesting that PGP 9.5 and TrpV1 play different roles in pain signal transmission. In our present study, PGP9.5 was not significantly different among the 4 groups in colorectum, spinal cord and hypothalamus, suggesting that PGP9.5 is not involved in zymosan-induced noninflammatory colorectal hypersensitivity and in the processing of acupuncture analgesia.

### 4.6. Possible Roles of p-ERK1/2 and TrpV1 in Zymosan-Induced Visceral Hypersensitivity and EA-Induced Analgesia in IBS

 Activation of theERKsignaling pathway in the periphery is likely necessary for the maintenance of a spinally sensitized state, while activation of ERK1/2 in the primary injury site may regulateTrpV1, leading to dorsal hornhypersensitivityto thermal and chemical stimuli [34]. Activation of ERK in primary afferent neurons is mediated, at least in part by TrpV1 [35]. In agreement with these two studies, our result shows that both p-ERK1/2 and TrpV1 in the periphery are involved in zymosan-induced hypersensitivity and EA-induced analgesia in IBS. However, in central nerves system, we detected a strong signal of p-ERK1/2 in response to zymosan treatment and this signal was greatly blunted by EA treatment, while TrpV1 was undetectable, suggesting that p-ERK1/2 is more important than TrpV1 in central nerves system in those processes. Further studies by using either ERK or TrpV1 knockout mice are necessary to clarify their correlation in the mechanism of EA in the future.

## 5. Conclusions

 EA significantly compromises zymosan-induced colorectal hypersensitivity, and the effectiveness of acupuncture analgesia is accumulative with increased number of times of acupuncture when compared to that of a single time of acupuncture. Ion channel, TrpV1 expression in colorectal, and ERK1/2 MAPK pathway activation in peripheral and central nerve system might be involved in this process. These results suggested that EA is a potential therapeutic tool to treat the abdominal pain or discomfort. 

## Abbreviations

CRD: Colorectal distension
EA: Electroacupuncture
IBS: Irritable bowel syndrome
VMR: Visceral motor response
EMG: Electromyographic.
